# Supplementary figures and images for: DANGER analysis: risk-averse on/off-target assessment for CRISPR editing without a reference genome
Source: Bioinform Adv. 2023 Aug 23;3(1):vbad114. doi: 10.1093/bioadv/vbad114 (PMC10469126; doi:10.1093/bioadv/vbad114)

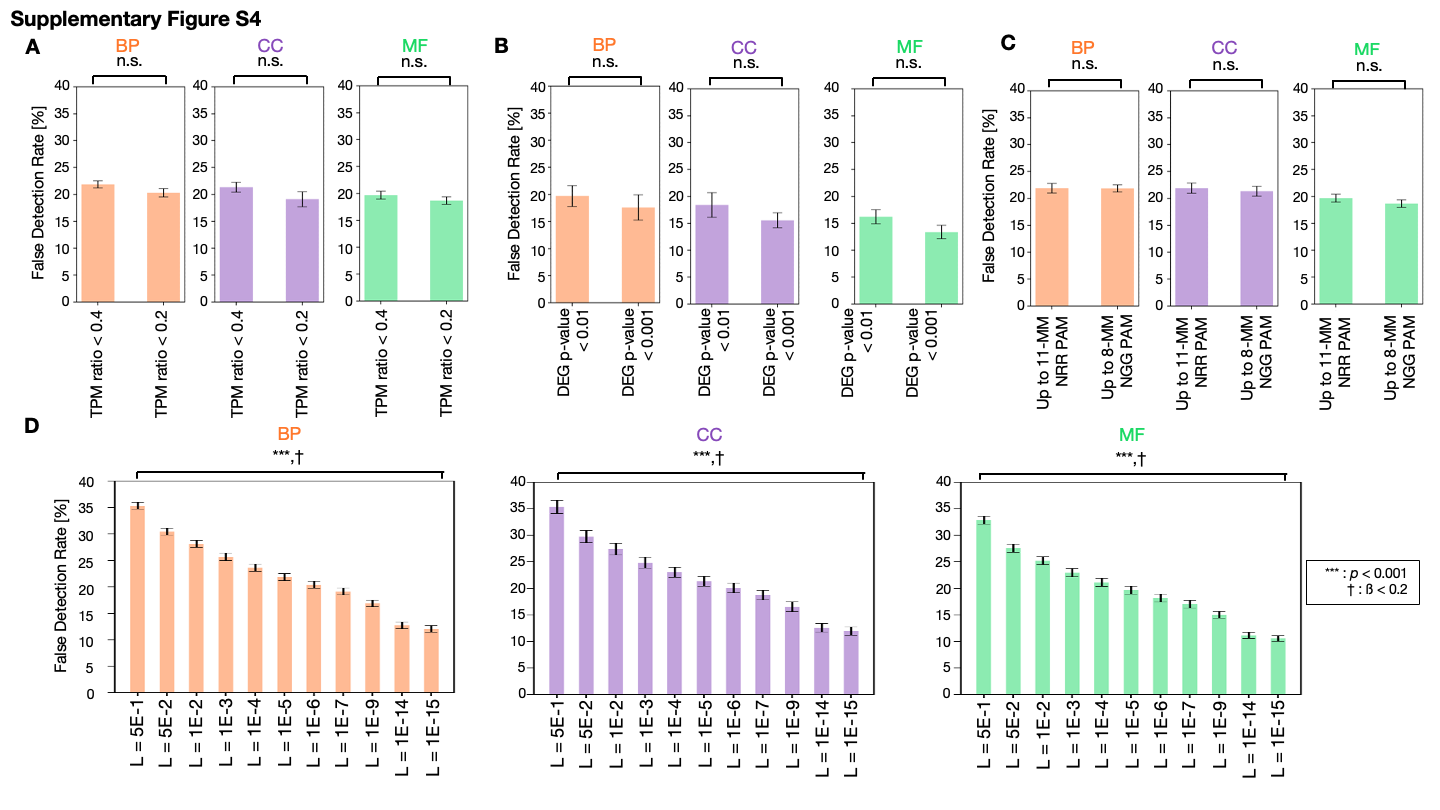

Supplement: vbad114_Supplementary_Data [file vbad114_supplementary_data.zip › Supplementary Figure S4.tiff]

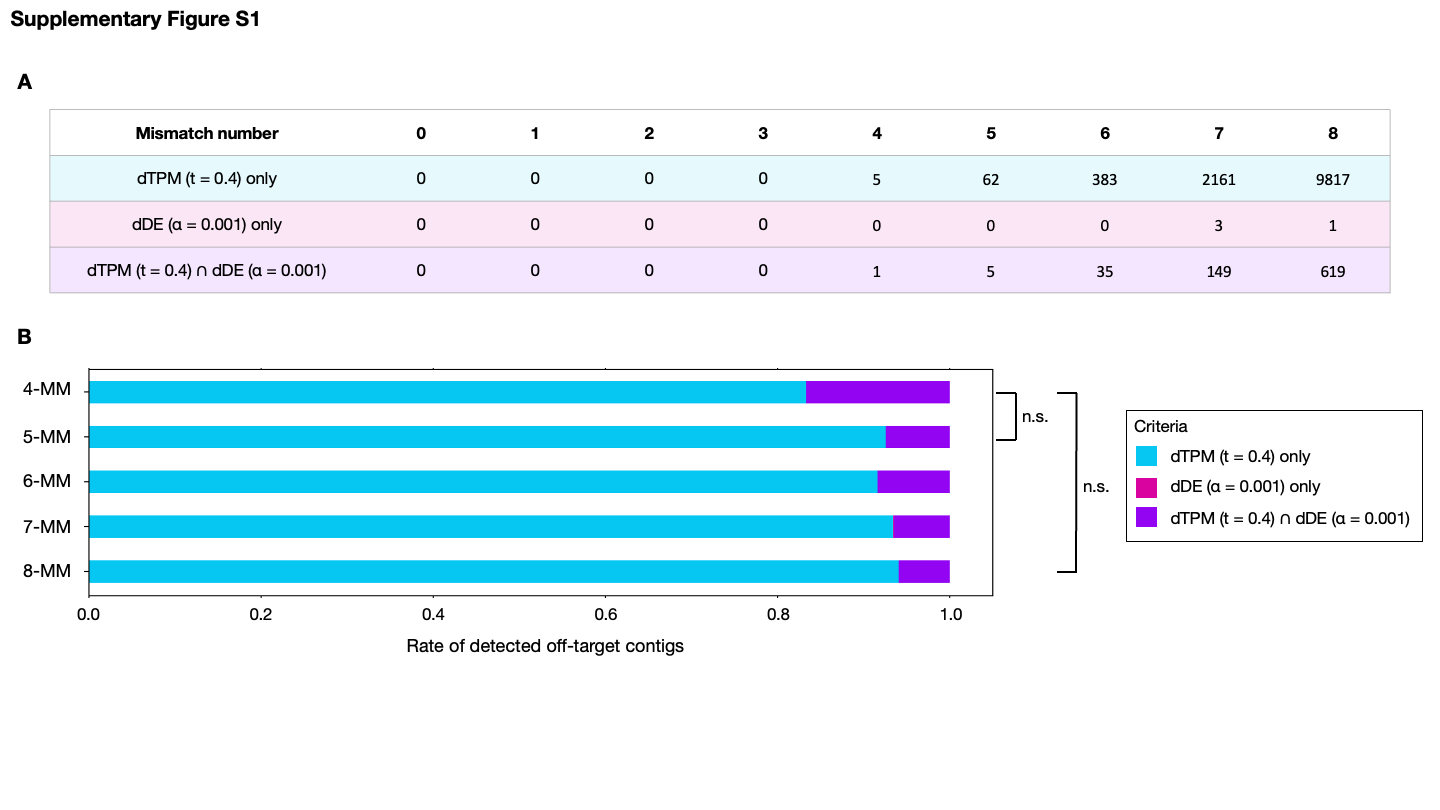

Supplement: vbad114_Supplementary_Data [file vbad114_supplementary_data.zip › Supplementary Figure S1.tiff]

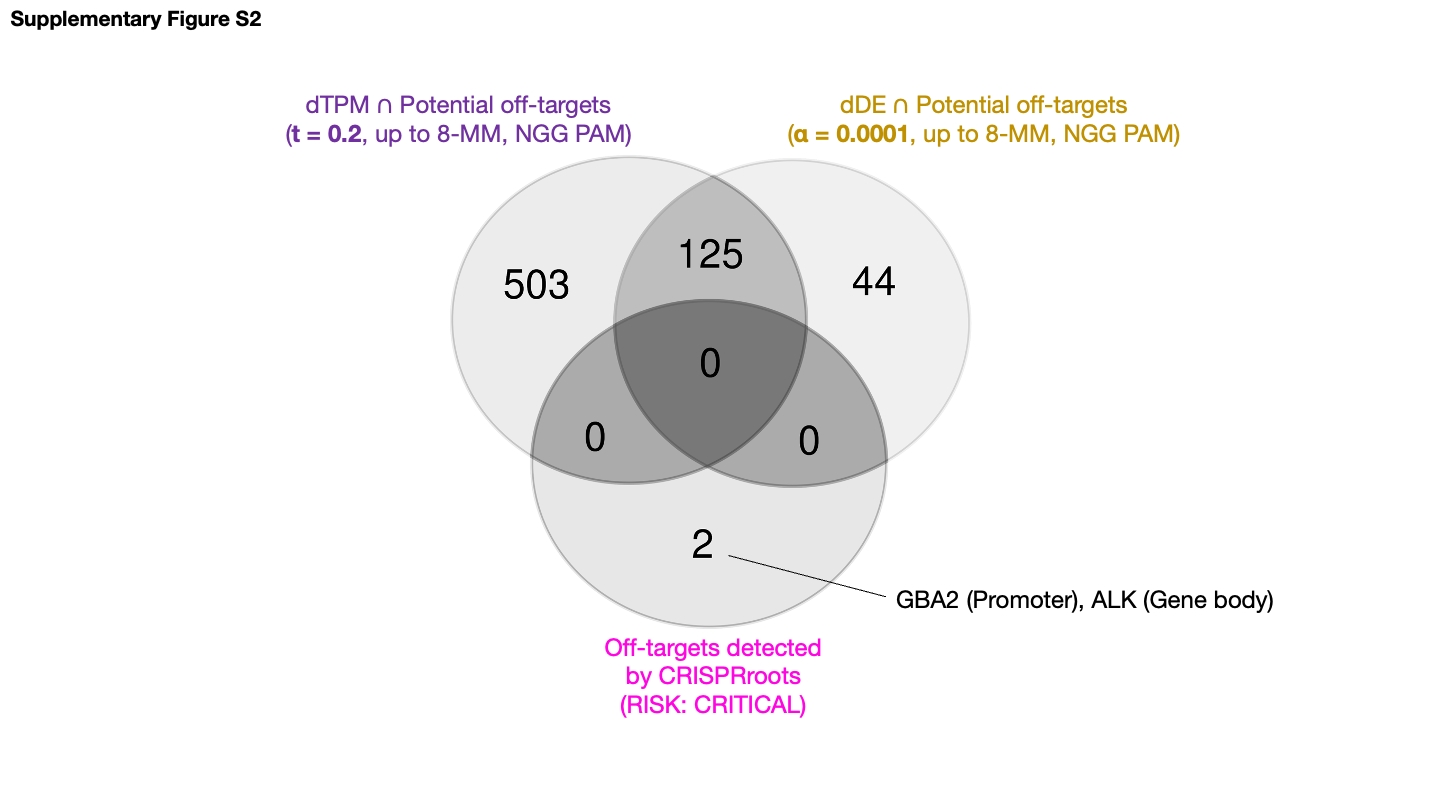

Supplement: vbad114_Supplementary_Data [file vbad114_supplementary_data.zip › Supplementary Figure S2.tiff]

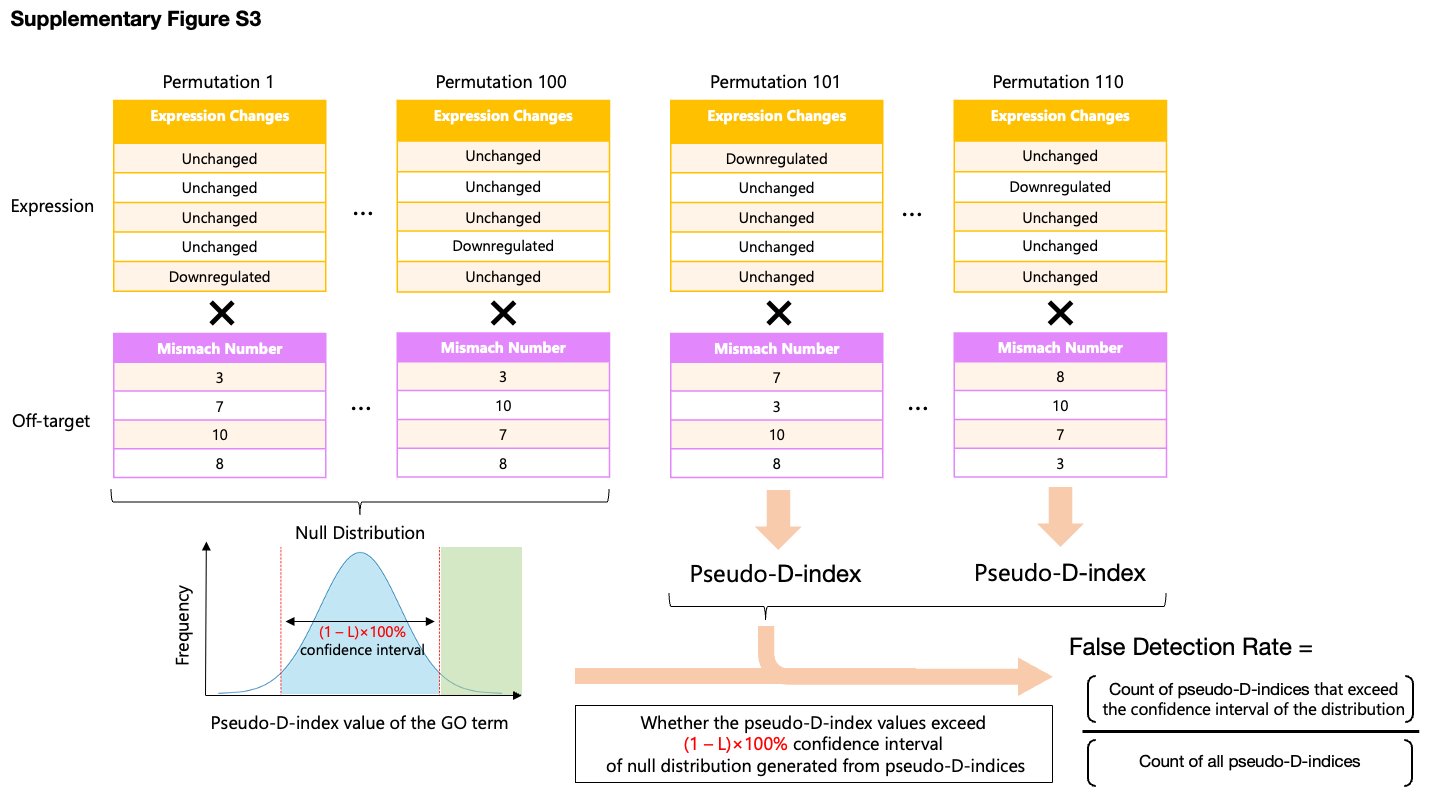

Supplement: vbad114_Supplementary_Data [file vbad114_supplementary_data.zip › Supplementary Figure S3.tiff]
